# Not All Parafibromin Deficiency Relates to Parathyroid Carcinoma: The Role of Morphological Assessment

**DOI:** 10.1007/s12022-024-09804-5

**Published:** 2024-02-16

**Authors:** C. Christofer Juhlin

**Affiliations:** Department of Pathology and Cancer Diagnostics, Karolinska University Hospital, Radiumhemmet P1:02, 176 64 Stockholm, Sweden

**Keywords:** Parathyroid tumor, Parafibromin, CDC73, Adenoma

## Case History

A male in his early thirties, without any prior medical history, presented with a history of fatigue. Upon examination, mild hypercalcemia and elevated levels of parathyroid hormone (PTH) were observed. The patient was subsequently referred to our endocrine surgery department. Ultrasound and sestamibi scintigraphy revealed an enlarged right superior parathyroid gland measuring 20 mm. The patient had no family history suggestive of parathyroid disease or MEN1-related manifestations. A focused parathyroidectomy was performed.

## What Is Your Diagnosis?

**Fig. 1:**
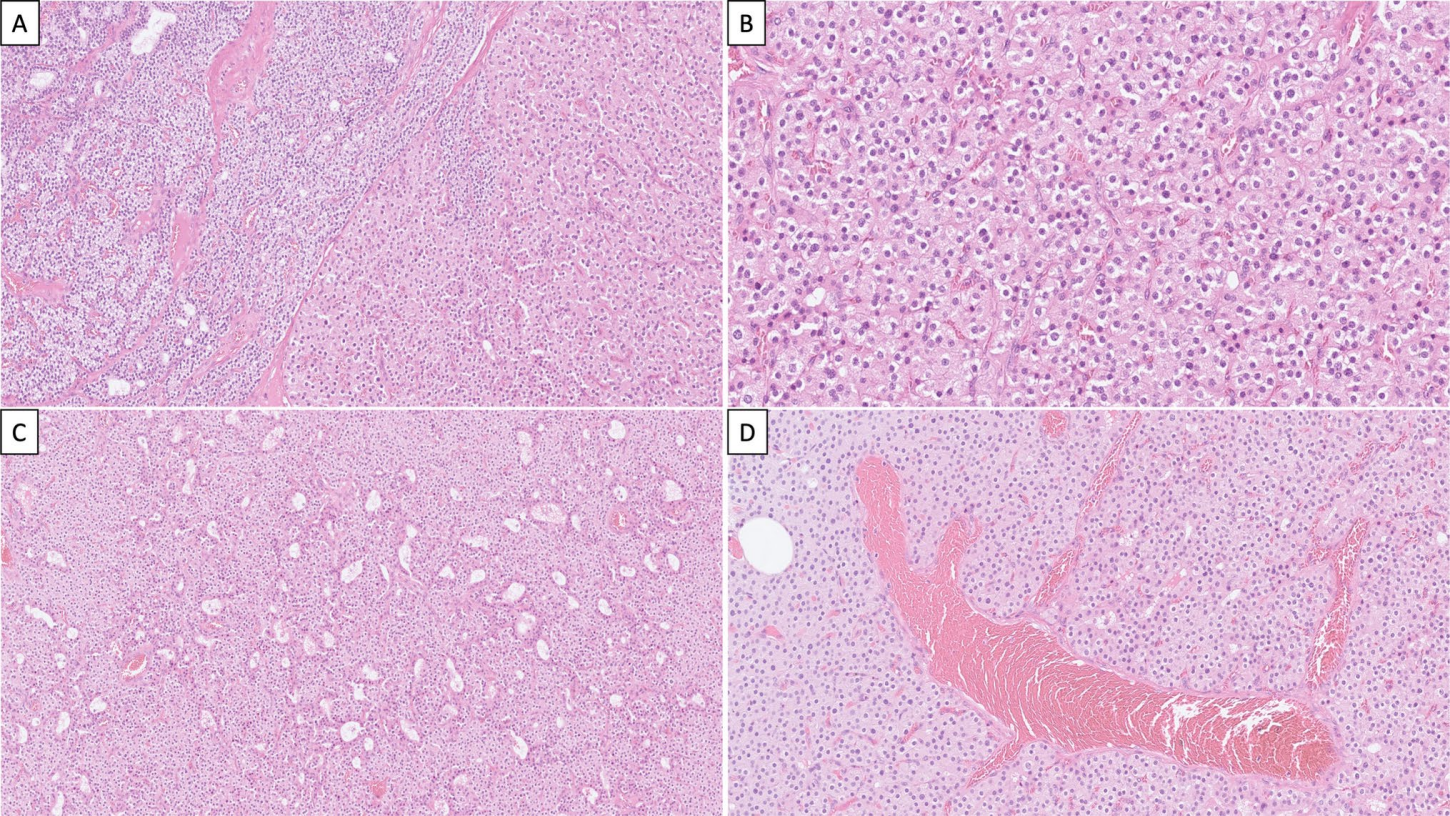
Morphological indicators of parafibromin deficiency in a parathyroid tumor. **A** This hypercellular parathyroid gland was composed of eosinophilic cells (right) intermingled with focal areas with chief cells (left). Note the absence of stromal fat. There were no atypical histological features associated to malignancy. **B** Eosinophilic cells exhibited perinuclear clearing. **C** Focal microcystic change. **D** Areas with arborizing vasculature within the tumor tissue are a recurrent finding in parafibromin-deficient parathyroid tumors

**Fig. 2:**
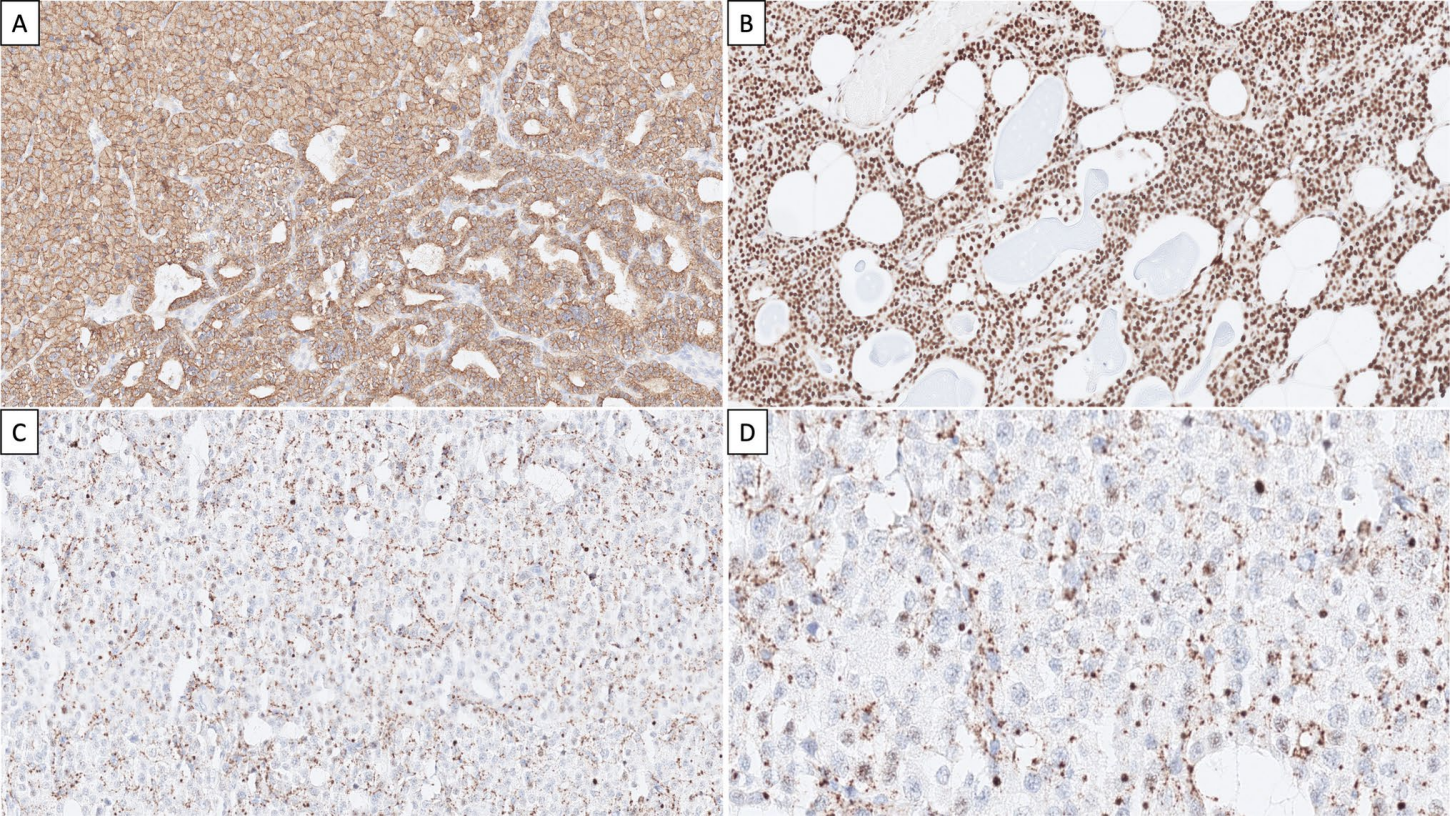
Immunohistochemical staining outcome. **A** PTH immunoreactivity was diffusely positive, thereby confirming the parathyroid origin and indicating the retained antigenicity of the sample. **B** Strong nuclear expression of parafibromin was evident in the normal parathyroid rim. **C** Eosinophilic tumor cells were parafibromin-deficient, exhibiting negative nuclear staining along with a granular cytosolic stain, interpreted as aberrant (× 100 magnification). **D** High-power (× 400) magnification of the parafibromin stain

Figure composites (see Figs. 1 and 2).

## Diagnosis: Parafibromin-Deficient Parathyroid Adenoma

During gross examination, the weight was 1.1 g. The gland measured 20 × 15 × 7 mm and displayed a brownish cut surface. No macroscopic features indicative of malignancy, such as a grey/white cut surface or unclear relations to the surroundings, were observed. The entire tumor was submitted for microscopic evaluation.

Histology revealed a hypercellular parathyroid gland characterized by chief cells arranged in microacinar patterns and solid areas containing eosinophilic cells (Fig. 1A). The stromal fat content was diminished. Notably, there was a general absence of mitotic activity and tumor necrosis. The tumor was circumscribed, and invasive growth was not observed. No atypical histological features such as trabecular growth, fibrous bands, or prominent nuclear pleomorphism were noted. Upon closer examination, the eosinophilic cells exhibited a distinct pinkish cytoplasm but lacked the characteristic cytoplasmic granularity of oxyphilic cells (Fig. 1B). Additionally, these cells showed perinuclear cytoplasmic clearing (“halo”). Some regions also displayed microcystic features and arborizing vasculature (Fig. 1C, D). These morphological features have been associated with parafibromin-deficient parathyroid tumors [1]. Tumor cells were positive for PTH and APC but negative for galectin-3 and PGP9.5 (Fig. 2A). The Ki-67 index was low (2.4%). Parafibromin exhibited positive staining in cells at the tumor margin and in the majority of chief cells (Fig. 2B). However, the nuclear stain was negative in the eosinophilic cell component, displaying a vague, granular cytosolic stain, interpreted as aberrant (Fig. 2C, D).

The favored diagnosis was a parafibromin-deficient parathyroid adenoma, and the patient was found to harbor a pathogenic *CDC73* gene mutation involving the nuclear localization signal of parafibromin.

## Comment

When pathologists discuss parafibromin, it is typically in the context of diagnosing an atypical parathyroid tumor or parathyroid carcinoma, as these conditions are more frequently associated with *CDC73* gene mutations compared to parathyroid adenomas [2, 3]. However, this case illustrates how morphological indicators can guide pathologists to order relevant immunohistochemical stainings, assisting the clinical team in identifying a potential syndromic case even in a benign scenario. In our clinical practice, immunohistochemistry is not routinely applied to screen parathyroid adenomas. Recognizing sheet-like eosinophilic tumor cells with a perinuclear halo and associated arborizing vasculature could therefore be important, offering endocrine pathologists a valuable genotype–phenotype correlation [1].

Speculatively, the identified *CDC73* mutation could potentially disrupt the nuclear localization of parafibromin, leading to its sequestration in the cytosolic compartment. Previous studies have demonstrated a crucial role for nuclear parafibromin to function as a tumor suppressor, implying that *CDC73* mutations in the NLS sequence could be pathogenic [4]. Similarly, parafibromin possesses a nucleolar localization signal (NoLS), and the loss of nucleolar parafibromin has been linked to *CDC73* mutations within the NoLS region [5]. Therefore, meticulous microscopic examination at high magnification is essential when evaluating parafibromin.

## Data Availability

The author confirms that the data supporting the findings of this study are available within the article.
